# Intravitreal aflibercept following treat and extend protocol versus fixed protocol for treatment of neovascular age-related macular degeneration

**DOI:** 10.1186/s40942-021-00349-x

**Published:** 2021-12-07

**Authors:** Alaa Din Abdin, Asem Mohamed, Cristian Munteanu, Isabel Weinstein, Achim Langenbucher, Berthold Seitz, Shady Suffo

**Affiliations:** 1 Department of Ophthalmology, Saarland University Medical Center UKS, Homburg/Saar, Germany; 2 Department of Ophthalmology, Westpfalz Hospital, Kaiserslautern, Germany; 3 Institute of Experimental Ophthalmology, Saarland University, Homburg/Saar, Germany

**Keywords:** Neovascular age-related macular degeneration, Aflibercept, Treat and extend, Fixed protocol

## Abstract

**Background:**

To assess the morphological and functional outcome of intravitreal aflibercept following the treat and extend protocol compared to the fixed protocol for treatment of eyes with neovascular age-related macular degeneration.

**Methods:**

This retrospective study included 126 eyes of 113 patients with primary onset neovascular age-related macular degeneration who were followed for 12 months. All eyes were treated with 2 mg/0.05 mL aflibercept. All eyes received an upload with three monthly aflibercept injections. We subsequently studied two groups of eyes. For group 1, 54 eyes were treated following the treat and extend protocol. For group 2, 72 eyes were treated following the fixed protocol (fixed 2-monthly interval). Main outcome measures included: best corrected visual acuity (BCVA), central macular thickness (CMT) and number of injections.

**Results:**

BCVA (logMAR) in group 1 vs group 2 was (0.61 ± 0.3 vs 0.72 ± 0.3, p = 0.09) before treatment and (0.48 ± 0.3 vs 0.51 ± 0.3, p = 0.6) after one year of treatment. CMT in group 1 vs group 2 was (371 ± 101 μm vs 393 ± 116 μm, p = 0.5) before treatment and (284 ± 60 μm vs 290 ± 67 μm, p = 0.1) after one year of treatment. Number of injections/eye in group 1 vs group 2 was (8.5 ± 2.2 vs 7.0 ± 0, p < 0.001).

**Conclusions:**

Significant differences regarding BCVA and central macular thickness were not found between both treatment protocols during the first year of treatment using aflibercept. However, a significantly higher number of injections was needed for eyes in the treat and extend group during the first year of treatment. This might suggest that aflibercept should better not be extended past an 8 weeks interval during the first year of treatment.

**Study registration:**

This study was approved by the Ethics Committee of the Medical Association of Saarland, Germany (Nr. 123/20, Date: 16.06.2020). All procedures performed in studies involving human participants were in accordance with the ethical standards of the institutional and/or national research committee and with the 1964 Helsinki declaration and its later amendments or comparable ethical standards. This article does not contain any studies with animals performed by any of the authors.

## Background

Age-related macular degeneration (AMD) affects the quality of life for elderly people and represents a major challenge for patients and health systems in developed countries [1].

Many medical reports have confirmed the importantance of the vascular endothelial growth factor (VEGF) in the development of choroidal neovascularization (CNV) in neovascular AMD [2]. It induces angiogenesis, increases vascular permeability, causes blood-retinal barrier breakdown and promotes an inflammatory response [3].

To date, three anti-VEGFs have been approved in Europe and the USA for the treatment of neovascular AMD: ranibizumab, aflibercept and brolucizumab [4, 5]:

Aflibercept (Eylea) was approved in 2011 for the treatment of neovascular AMD. It is a recombinant fusion protein that strongly binds to VEGF and placental growth factor, and inhibits the binding and activation of the cognate VEGF-receptors [6, 7]. The intravitreal half-life of aflibercept is 9.0 days [8].

Aflibercept was primarily administered following the fixed protocol supported by VIEW 1 and VIEW 2 studies, with injections every 2 months following an upload phase with 3 monthly injections [9].

However, a number of flexible treatment protocols, such as pro re nata (PRN) and treat and extend (T&E), have increasingly been used in clinical practice in order to avoid any under treatment [10, 11]. The T&E protocol suggests fixed treatment decisions in variable retreatment intervals according to the clinical course [11].

Some clinical studies reported several advantages for the T&E protocol over other treatment protocols especially after the first year of treatment, including better stability of disease, better patient compliance and better organization of surgical schedules [11]. However, it is still not clear in real life experience whether the T&E protocol is suitable in the first year for treatment of neovascular AMD with aflibercept and how it does affect the morphological and functional outcome compared to the fixed regimen.

Aflibercept was initially administered following the fixed protocol in our department. As clinical studies reported several advantages for the T&E protocol [11]. We gradually changed our practice.

The purpose of the present study is to assess the morphological and functional outcome of intravitreal aflibercept following the treat and extend protocol compared to the fixed protocol for the treatment of eyes with neovascular age-related macular degeneration.

## Materials and methods

### Description of study groups

This retrospective study included 126 eyes of 113 patients with primary onset neovascular AMD who were followed up for 12 months.

All eyes were treated with 2 mg/0.05 mL aflibercept (Eylea, Bayer Pharma AG, Berlin, Germany). All eyes received an upload with three monthly aflibercept injections.

Then, we studied two groups of eyes:

- Group 1: 54 eyes were treated following the treat and extend protocol.
- Group 2: 72 eyes were treated following the fixed protocol (fixed 2-monthly interval).

Injections were performed in a designated intravitreal injections center in our Department of Ophthalmology at Saarland University Medical Center [12].

The inclusion criteria were

1. Symptomatic primary onset neovascular AMD (CNV types 1 and 2).
2. Three consecutive monthly aflibercept injections.
3. A minimum follow-up of 12 months.

The exclusion criteria were

1. History of treatments, including photodynamic therapy (PDT) or any previous intravitreal injection.
2. Eyes with massive hemorrhages or advanced fibrosis.
3. Eyes with CNV type 3.
4. Intraocular surgeries (Cataract surgery, pars plana vitrectomy) during the first year of treatment.

For group 1, eyes were treated with a treat and extend algorithm; the treatment interval was gradually extended by 2 weeks at a time up to a maximum of 12 weeks as long as no signs of activity were seen.

However, it was reduced by 2 weeks if a minor recurrence, defined as presence of mild intraretinal or subretinal fluid without visual loss or foveal hemorrhage, was evident. The treatment interval was reverted to monthly treatments if a major recurrence, defined as presence of severe intraretinal or subretinal fluid associated with visual loss > 6 letters and/or presence of foveal hemorrhage, was evident [11].

Main outcome measures: included:

- Best corrected visual acuity (BCVA).
- Central macular thickness (CMT) as measured by Spectral Domain Optical Coherence Tomography (Spectralis SD-OCT; Heidelberg Engineering, Heidelberg, Germany).
- The number of injections during the first year of treatment.

### Statistics

A kruskal wallis test was performed to check for normal distribution. A Mann–Whitney-U test (nonparametric statistics) was performed to examine the effect of time (before and after treatment) and group on BCVA. A two-way ANOVA was conducted to examine the effect of time (before and after treatment) and group on CMT and number of injections. Data were presented as mean ± standard deviation. Results were considered statistically significant if p values < 0.05.

## Results

The patients’ baseline characteristics in both groups are summarized in (Table 1).

**Table 1:** Baseline characteristics of the study groups (means ± SD)

Variable	T&E protocol (n = 54)	Fixed protocol (n = 72)	p-value
Gender (Male:Female)	43%:57%	36%:64%	0.3
Eye (Right:Left)	52%:48%	48%:52%	0.5
Patient age (years)	80 ± 7	81 ± 6	0.6
CNV type (1:2)	56%:44%	58%:42%	0.7
Phakic:Pseudophakic	27%:73%	13%:87%	0.9
BCVA (Log MAR)	0.61 ± 0.3	0.72 ± 0.3	0.09
CMT (μm)	371 ± 101	393 ± 116	0.5

T&E: Treat and extend, CNV: Choroidal neovascularization, BCVA: Best corrected visual acuity, CMT: Central macular thickness

p value refers to statistical differences between two groups

### BCVA

BCVA in group 1 vs group 2 was 0.61 ± 0.3 vs 0.72 ± 0.3 (p = 0.09) before treatment and 0.48 ± 0.3 vs 0.51 ± 0.3 (p = 0.6) after one year of treatment.

**Fig. 1:**
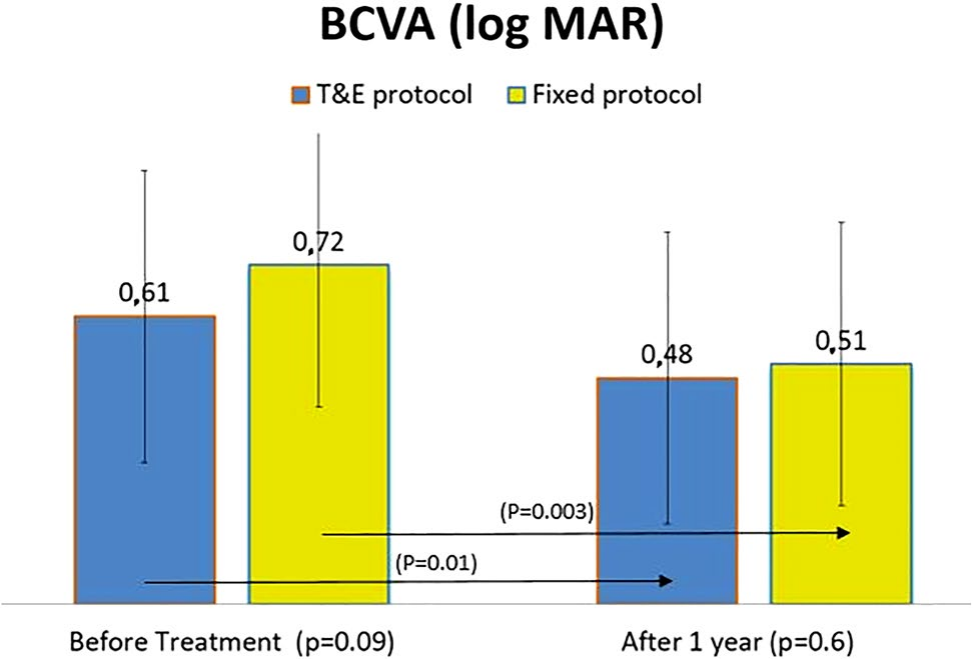
There was no statistically significant difference regarding BCVA (Log MAR) between both groups before and after treatment. Results are given as means ± standard deviation

The visual improvement (decimal) after one year was statistically significant without statistically significant differences between both groups 0.1 ± 0.1 vs 0.14 ± 0.1 (p = 0.1) (Fig. 1). The BCVA improvement (approximate ETDRS letter scores) in group 1 vs group 2 was 7 ± 11 vs 10 ± 13 (p = 0.1).

### CMT

**Fig. 2:**
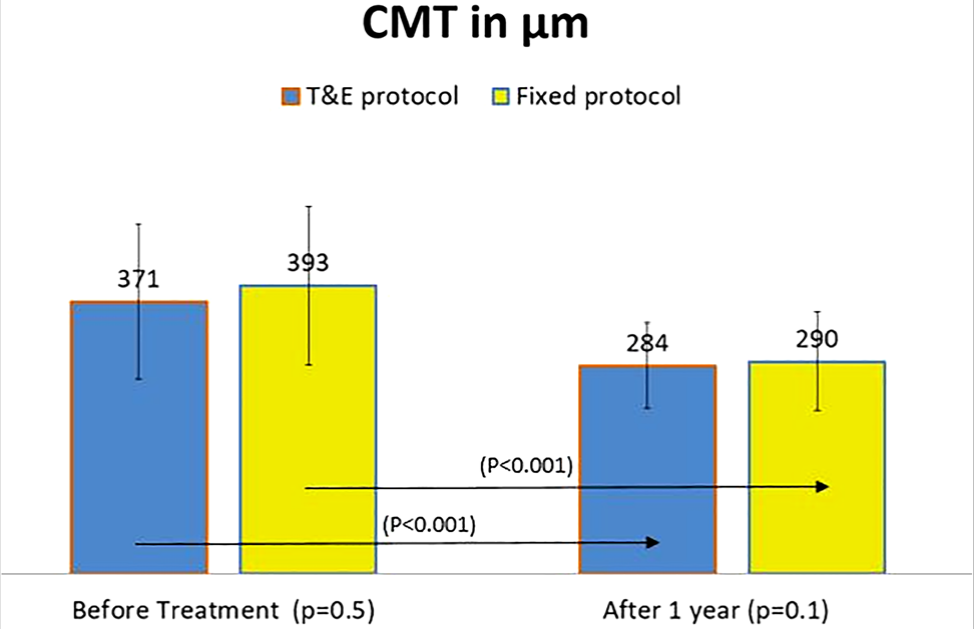
There was no statistically significant difference regarding mean CMT between both groups before and after treatment. Results are given as means ± standard deviation

CMT in group 1 vs group 2 was 371 ± 101 μm vs 393 ± 116 μm (p = 0.5) before treatment and 284 ± 60 μm vs 290 ± 67 μm (p = 0.1) after treatment (Fig. 2). The decrease in CMT after one year was statistically significant without statistically significant differences between both groups 76 ± 102 μm vs 102 ± 110 μm (p = 0.1).

### The number of injections/eye

The number of injections/eye in group 1 vs group 2 was 8.5 ± 2.2 vs 7.0 ± 0 (p < 0.001). The mean number of visits/patient in group 1 vs group 2, including the first visit with upload phase, was 9.8 ± 1.4 vs 8.0 ± 0 (p < 0.001).

In group 1, 21 eyes were extended to 12 week intervals without recurrences. However, minor recurrences were evident 27 times in 22 eyes and major recurrences were evident 13 times in 13 eyes. (Table 2).

**Table 2:** Recurrences in the T&E group

Recurrences	After 6 weeks interval	After 8 weeks interval	After 10 weeks interval	After 12 weeks interval	Total
Minor changes	5	5	9	2	27
Major changes	2	3	8	0	13

T&E: Treat and extend

## Discussion

In this study, we found no significant differences between our two groups in baseline characteristics: Patient age, patient gender, CNV type, BCVA, and CMT before treatment, which gave us the opportunity to investigate whether the T&E protocol and the fixed protocol are equally effective after 12 months for treating neovascular AMD with aflibercept.

Regarding BCVA, there was no significant difference between the two groups after 12 months. The mean CMT was also comparable between the two groups after 12 months. Furthermore, both compared protocols were equally effective in improving BCVA and decreasing CMT at 12 months.

One of the most important challenges related to the treatment of neovascular AMD with anti-VEGF agents is the frequency of injections in real-life settings, which remain unstable most of the time [13]. In the T&E protocol, the patient receives an injection every visit, which provides a relatively stable treatment plan and consequently better stability of disease [11]. In this study, patients in the T&E group received an average of 8.5 injections in the first year of treatment. This was significantly higher than the mean number of injections in the fixed protocol group (7 injections in the first year).

Our results could be supported by the VIEW studies, which reported a mean number of 7.0 to 7.5 active aflibercept injections during the first year of treatment following the fixed protocol [9, 14].

However, the overall mean number of aflibercept injections during the first year of treatment following the T&E protocol was variable in the real-world data. Some studies reported between 7.2 and 7.7 injections for example [15–18], whereas other studies reported between 9 and 9.7 injections in the first year of treatment [19, 20]. This difference could be related to the retreatment criteria, which were not strictly applied in all studies. In our study, we followed relatively strict retreatment criteria compared to other studies. In the present study, 22 eyes in the T&E group had minor changes and the treatment interval was reduced by 2 weeks, whereas 13 eyes had major changes reflecting re-activity with reduced visual acuity and/or foveal hemorrhage and the treatment interval was reverted to monthly treatments. This might clarify the significantly higher number of injections in the T&E group.

In 2019, a previous study from our group reported a higher major recurrence rate in aflibercept compared to ranibizumab patients during the first year following an identical treat and extend protocol [19]. This could be supported by the results of the present study, especially when we notice that most major changes in the T&E group (8 from 13 eyes) were evident after extending the treatment interval to 10 weeks. Thus, we might suggest that aflibercept should not be extended past an 8 weeks interval during the first year of treatment, which was already recommended in the VIEW studies.

Main potential limitations of our study were the retrospective nature of the work, a relatively small population from a single medical center, relying upon Snellen VA as opposed to ETDRS vision charts, which are normally used in major clinical trials. However, for more reliable values to describe the BCVA improvement, the log MAR values were converted to letter score equivalents (Approximate ETDRS Letter Scores) using the formula “log MAR = 1.7-(0.02) (letter score)” [21]. With this conversion, a 5-letter difference in visual acuity is equivalent to a difference of 0.1 log MAR and to one Snellen line.

Finally, further studies with a longer follow-up period are required in this field to determine the best protocol for treatment of eyes with neovascular age-related macular degeneration in our real-world practice.

## Conclusions

Significant differences regarding BCVA and central macular thickness were not found between both treatment protocols during the first year of treatment with aflibercept. However, a significantly higher number of injections was needed for eyes in the treat and extend group during the first year of treatment. This might suggest that aflibercept should better not be extended past an 8 weeks interval during the first year of treatment.

## Data Availability

The datasets used and/or analysed during the current study are available from the corresponding author on reasonable request.

## Abbreviations

AMD: Age-related macular degeneration
BCVA: Best corrected visual acuity
CMT: Central macular thickness
CNV: Choroidal neovascularization
PDT: Photodynamic therapy
PRN: Pro re nata
T&E: Treat and extend
VEGF: Vascular endothelial growth factor
